# Microbial Interventions to Improve Neonatal Gut Health

**DOI:** 10.3390/microorganisms11051328

**Published:** 2023-05-18

**Authors:** Ranga Nakandalage, Le Luo Guan, Nilusha Malmuthuge

**Affiliations:** 1 Department of Agricultural, Food and Nutritional Science, University of Alberta, Edmonton, AB T6G 2P5, Canada; 2 Lethbridge Research and Development Center, Agriculture Agri-Food Canada, Lethbridge, AB T1J 4B1, Canada

**Keywords:** neonatal gut health, mucosal immune system, microbial interventions

## Abstract

The diverse pioneer microbial community colonizing the mammalian gastrointestinal tract is critical for the developing immune system. Gut microbial communities of neonates can be affected by various internal and external factors, resulting in microbial dysbiosis. Microbial dysbiosis during early life affects gut homeostasis by changing metabolic, physiological, and immunological status, which increases susceptibility to neonatal infections and long-term pathologies. Early life is crucial for the establishment of microbiota and the development of the host immune system. Therefore, it provides a window of opportunity to reverse microbial dysbiosis with a positive impact on host health. Recent attempts to use microbial interventions during early life have successfully reversed dysbiotic gut microbial communities in neonates. However, interventions with persistent effects on microbiota and host health are still limited. This review will critically discuss microbial interventions, modulatory mechanisms, their limitations, and gaps in knowledge to understand their roles in improving neonatal gut health.

## 1. Introduction

Mammalian gut health has gained research and public attention over the last decade due to its influence on overall host health [1,2]. Traditionally, gut health has been defined as the ability to maintain disease- or pathogen-free status [3]. However, gut health includes several physiological and functional features of the gastrointestinal tract (GIT) related to nutrient digestion and absorption, host metabolism, microbiota, barrier function, and mucosal immune responses [1,2,3]. Therefore, gut health can be defined as a multifactorial concept that depends on the host and microbial interactions to maintain metabolism, homeostasis, immune functions, and overall wellbeing.

Host–microbe interactions form one of the primary elements of gut health [4,5,6], which can be affected by various internal and external factors throughout life. Birth mode (vaginal delivery vs. cesarean delivery), gestational age (premature vs. full-term), antibiotic treatments, diet (breastfed vs. formula-fed; fiber-rich vs. high-sugar, high-fat diet), infections, habits (exercise, stress), and environmental factors (geographical region) have been linked to perturbed microbiota and microbiome-linked pathologies [7,8,9,10,11,12,13,14,15]. However, some of these factors that lead to microbial dysbiosis are life-saving medical interventions and are thus indispensable. For example, despite the vast literature supporting the presence of microbial perturbations in cesarean-delivered babies and the increased risk of developing microbiome-linked pathologies later in life, it has tremendously improved the survival of babies and moms by reducing the number of deaths due to complications during pregnancy [16]. In such situations, microbial interventions can be used to restore perturbed microbial communities and to mitigate negative health outcomes/microbiome-linked pathologies.

During early colonization, gut microbial community composition and diversity change rapidly. This rapidly evolving neonatal microbial community is less resilient when compared to an established adult microbiome, creating a crucial time window (window of opportunity) to use microbial interventions. In infants, the first 1000 days of life provide the highest efficacy to intervene and restore a perturbed microbial community [17]. With recent advances in identifying and defining the window of opportunity for microbial interventions, researchers have successfully created comparatively persistent changes in the gut microbial community of neonates. For example, vaginal seeding is a microbial intervention that introduces maternal microbiota to cesarean-delivered infants by exposing them to maternal vaginal content [18]. When cesarean-delivered infants received vaginal seeding at birth, their microbial communities closely resembled those of vaginally delivered infants [18,19]. Moreover, this changed the trajectories of gut microbial colonization in cesarean-delivered infants [19]. In this observational study, the researchers reported that their intervention altered both the oral and fecal microbial communities of infants, and the changes were persistent during the first year of life. Korpela and colleagues [20] partially restored the microbial communities of cesarean-delivered babies using maternal fecal matter transplantation. Another study used probiotic supplementation in preterm infants with extremely low birth weight and reported alterations in the gut microbial community at one month of age but not at two years [21]. All the evidence suggests that intervening in gut microbiota during the early life window can create persistent outcomes later in life. Therefore, there is a tremendous opportunity to develop successful intervention tools to alter host–microbial interactions to improve gut health.

During the window of opportunity, the neonatal immune system also undergoes significant changes in response to the pioneer gut microbiota [22]. The microbial priming of innate and adaptive immune systems initiates gut homeostasis by balancing protective and regulatory immune responses [23]. Therefore, gut microbial perturbations during early life can lead to long-term adverse health outcomes, and restoring the pioneer microbial community can reverse these effects [24]. As we mentioned earlier, early-life microbial interventions have been used to reverse microbial perturbations successfully [25,26]. However, microbial interventions with persistent effects on gut health are still limited. This review will critically discuss microbial interventions, modulatory mechanisms, their limitations, and gaps in knowledge to understand their roles in improving neonatal gut health.

## 2. Role of Pioneer Microbiota and Early Life Window in Neonatal Gut Health

The colonization of gut microbiota starts during the birthing process when the fetus is exposed to the outside following the rupture of amniotic membranes [27]. There are in-depth reviews on the factors that affect the composition of pioneer gut microbiota in infants [17,27] and microbial dysbiosis-related adverse health effects (microbiome-linked pathologies) during early life [28,29]. In addition to infants, recent studies on livestock have shown that early life events are linked to variations in the microbial community. For example, naturally born calves had a higher microbial richness, evenness, and diversity in the rumen when compared to cesarean-delivered calves [30]. Moreover, the time of colostrum feeding has been shown to affect the establishment of the microbial community in newborn calves [31]. In piglets, antibiotics have been reported to alter the gut microbiota community [32,33,34,35,36]. Moreover, piglets raised in isolators and treated with antibiotics were reported to have altered microbial communities and immune responses [37].

Maintaining a balance between protective and regulatory immune responses is vital for neonatal gut health. Neonates are particularly susceptible to infections, more so than adults, until their immune systems are functionally developed [23,38,39]. Germ-free animal models have revealed the causal relationship between commensal gut microbiota and the development of the host immune system during early life. For example, germ-free mice have fewer B cells [40,41,42] and lower levels of secretory antibodies and antimicrobial peptides when compared to conventional mice [40,43,44,45]. Moreover, studies have shown that preterm infants with perturbed gut microbial communities lack goblet cells, Paneth cells [46], and natural killer cells [47] compared to full-term infants [48,49]. This immune memory activated by the pioneer microbiota is known as immune imprinting [50]. When the pioneer microbial community is perturbed, the interactions between host and gut microbiota can lead to the overactivation of reactive immune responses, disturbing immune homeostasis [50,51]. However, these altered immune functions can only be returned by restoring microbiota during the neonatal period (birth to weaning) [44], indicating that microbial interventions to modulate the immune system should be conducted during this window of opportunity. In germ-free mice, the accumulation of invariant natural killer T cells (iNKT) in the colon and lung increased susceptibility to inflammatory bowel disease and asthma [52]. However, the colonization of germ-free mice before weaning (in the neonatal period) could only reduce iNKT numbers and disease incidence [52]. The recognition of lipid antigens (self and non-self) by iNKT cells, which establish a long-term residency in tissues, activates pro-inflammatory cytokines and skewed immune responses towards inflammation [53,54]. Inflammation is one of the key signatures of microbiome-linked pathologies, and early-life microbial colonization plays a vital role in maintain immune homeostasis by modulating the development of the immune system. Similarly, germ-free mice have fewer mucosal-associated invariant T cells (MAIT) than specific-pathogen-free mice [55]. When germ-free mice were colonized during early life with a cocktail of commensal bacteria from wild-type mice, it restored the number of MAITs, but this was not the case in adult mice [55]. These studies suggest that immune imprinting occurs when introducing microbiota during the neonatal period but not after. Thus, the neonatal period is a crucial time window to intervene in the gut microbiota to maturate immune functions and ensure a healthy gut environment later in life.

In addition to priming balanced immune responses, gut microbiota also plays a vital role in maintaining gut epithelial barrier functions [56,57]. Appropriate regulation of barrier functions is another aspect of gut health [3]. High intestinal permeability is also common in preterm infants due to their immature gut microbiota composition [58]. However, *Bifidobacterium breve* and human milk oligosaccharides decrease intestinal permeability in preterm infants [58]. A recent study in piglets revealed that antibiotic treatments for diarrhea decrease gut permeability [59]. In this study, ampicillin administration for three days decreased the relative expression of tight-junction and adherence-junction proteins in the colons of newborn piglets. The expression of these proteins increases following fecal microbial transplantation (FMT), indicating that microbial restoration improves barrier functions. In neonatal calves, feeding with colostrum improves the gut barrier integrity compared to feeding with formula by increasing the expression of tight-junction proteins at four days of age [60]. Feeding with colostrum has also been shown to increase the colonization of beneficial bacteria such as Lactobacillus and Bifidobacterium in the GIT of newborn calves [9,61,62]. However, knowledge is lacking regarding whether the beneficial changes in the gut microbial community due to colostrum feeding play a role in the improved barrier functions of calves. Neonatal calf diarrhea is one of the major concerns in dairy calves, as it increases gut permeability [63] and alters gut microbial community [64] Recently, FMT has been used to minimize neonatal calf diarrhea and to improve gut health in pre-weaned calves [64,65,66]. These studies reported that FMT successfully altered gut microbial community. However, the impact of FMT on intestinal barrier functions and gut permeability is yet to be understood. The use of probiotic supplements in dairy calves has been shown to increase the expression of tight-junction gene zonula occludens-1 and occludin, while increasing microbial diversity [67]. Therefore, studying the impact of FMT on gut barrier functions will explain the modulatory mechanisms behind reduced diarrhea in dairy calves during FMT. These studies highlight the importance of beneficial pioneer microbiota in regulating intestinal permeability. A well-maintained gut barrier is crucial for neonates to maintain gut homeostasis. Thus, it is evident that the pioneer microbiota plays a vital role in maintaining gut health by modulating immune functions, cellular populations, and barrier integrity.

## 3. Role of Gut Microbiota in Vaccine Efficacy and Effectiveness

Immunization programs for infants and toddlers minimize neonatal infections by inducing antibody-dependent immune responses. For example, the oral rotavirus vaccine (ORV) minimizes the global burden of infant diarrhea among children under five in low- and middle-income countries by preventing 13.6 million illnesses that require antibiotic treatments [68,69]. Although vaccines are an effective preventative measurement, vaccine efficacy (the ability of vaccines to generate antibody responses) is highly inconsistent among different populations [69,70]. In particular, vaccine efficacy varies depending on socio-economic status: vaccines given to children in low- and middle-income countries show lower efficacy than those administered in high-income countries [69]. Assessment of the efficacy of ORV among different countries revealed that vaccine efficacy is high in USA and Europe compared to that in Bangladesh, Malawi, and Ghana, indicating a significant difference in the protection level depending on socio-economic status [70]. Lynn and colleagues [69] presented comprehensive details on studies reporting how gut microbial communities and immune responses vary between these two socio-economic statuses, while generating a hypothesis to link gut microbial communities to vaccine efficacy. In addition, few other critical recent reviews assess the roles of gut microbiota, probiotics, prebiotics, and antibiotics in vaccine efficacy [71,72,73]. Countries with different socio-economic statuses also have significantly different dietary/nutrition patterns, a key factor that affects gut microbial composition [74]. Therefore, we propose that the impact of socio-economic status on vaccine efficacy needs further investigation to dissect the roles of nutrition and gut microbiota. A recent study conducted by De Koff and colleagues [75] revealed that immune responses to vaccines vary between vaginal-delivered and cesarean-delivered infants. Vaginal-delivered infants generated higher antibody responses to pneumococcal and meningococcal vaccination than those born via cesarean section surgeries [75]. When the authors used gut microbial stability (beta-diversity between two consecutive sampling time points) as an indicator to assess the association between microbial community and vaccine responses, microbial stability during the first two weeks was positively correlated to antibody responses to the pneumococcal vaccine. The delivery mode has the highest impact on the gut microbial community during the first two weeks of life [76], indicating that differences in vaccine responses might have been attributed to variations in gut microbial communities.

While studies on humans provide knowledge on potential associations, animal models present direct evidence of the causal relationship between microbiota and immune responses, as well as between microbiota and nutrition. The use of antibiotics to treat both dams and pups reduced the density of total bacteria and phyla Firmicutes and Bacteroidetes in mice pups [43]. When the immunoglobulin levels of these mice pups were evaluated following immunization, antibiotic-treated mice pups with lower bacterial densities generated significantly lower immunoglobulin responses than their conventional counterparts. Similarly, early-life exposure to antibiotics in mice has been shown to produce a reduction in bacterial density, diversity, and antibody responses to vaccination compared to mice that did not receive antibiotics [77]. Animal models have also been used to assess the impact of nutrition on vaccine effectiveness in neonates [78,79]. A study conducted on mice with different protein diets revealed that the supplementation of low-protein diets results in significantly lower virus-specific antibodies and CD8^+^ T cells against influenza infections compared to mice supplemented with adequate protein diets [78]. When germ-free piglets receiving varying nutrition levels were immunized with the rotavirus vaccine, the malnourished piglets had significantly lower immunoglobulin G (IgG) in their serum than nourished piglets [79]. The transplantation of infant fecal microbiota to germ-free piglets before vaccination increased IgG responses in malnourished piglets more than those of germ-free malnourished and nourished piglets. The highest immune responses were observed in the colonized, nourished piglets [79], suggesting that gut microbes and nutrition work in synergy to increase host responses to vaccines. 

Vaccination is a cost-effective management practice in livestock production systems to minimize infections and antimicrobial treatments. However, the roles of gut microbiota and diet/nutrition have not been considered when designing the existing vaccination protocols. In addition, early vaccination has the greatest benefit via priming the developing immune system when it is designed to avoid interference from maternal antibodies [80,81,82]. The use of intranasal vaccines to prime neonatal immunity has become an important aspect of neonatal calf vaccination [83]. Maternal antibodies can successfully neutralize the modified live viruses in injectable vaccines, failing to prime immune responses in pre-weaned calves. Intranasal vaccines are effective in initiating short-duration protective responses in neonatal calves in the face of maternal antibodies [84], while inducing some priming of the long-term immunity [85]. Therefore, future research is necessary to maximize efficacy and effectiveness by incorporating microbiome analysis into vaccine trials. Moreover, it is vital to understand the role of restoring perturbed microbial communities before vaccinating neonates. The diet of livestock species is mainly designed based on the requirements for growth and metabolism. However, diet/nutrition affects the gut microbial community, which plays a role in immune imprinting and host responses to vaccines. Although microbial interventions are extensively studied in animals to improve health and production, future research (microbial interventions) needs to assess the immune response to vaccination to confirm that these improvements also benefit the animals by increasing the effectiveness of vaccines.

## 4. Potential Use of Microbial Interventional Tools to Improve Neonatal Gut Health

Globally, countries have launched antimicrobial stewardship programs to promote the fair use of antimicrobials in livestock production systems and human medicine. With this movement, the value of microbial interventions that promote gut health has received much appreciation from research, industry, and the public. Microbial interventions, such as probiotics, prebiotics, synbiotics, and postbiotics, are among the most highly anticipated candidates for maintaining healthy gut microbial communities that promote gut health [86]. Research in human and other animal models has focused on health status (number of days of hospitalization and incidence of diarrhea) during the supplementation of microbes and their substrates [87] to identify effective interventions. A longitudinal study that used metagenomic sequencing to study the fecal microbiota of very preterm infants (less than 32 weeks of gestation) reported that the supplementation of commercial probiotics (mixture of *Bifidobacterium* spp. and *Lactobacillus* spp.) altered fecal microbial communities by increasing the colonization of Bifidobacterium [88]. In contrast, the very preterm infants who did not receive probiotics were more likely to be colonized by *Klebsiella* spp.-enriched microbial communities [88]. Moreover, probiotics increased the diversity of the fecal microbiota [88], indicating that early-life microbial interventions influence the overall microbial community. A randomized, controlled intervention trial conducted on extremely preterm infants using a cocktail of probiotics (four Bifidobacterium strains and *Lacticaseibacillus rhamnosus*) revealed that intervention accelerates the establishment and maturation of the gut microbial community [89]. The administration of probiotics during the first week of life, followed by a two-week washout period, increased the colonization of Bifidobacterium in the infants at six months of age compared to those who had not received probiotics [89]. Interestingly, these comparatively persistent changes in the microbial community of the probiotics-treated group also coincided with a reduction in pro-inflammatory cytokines (IFNγ, Il-12, Il-4) and an increase in regulatory cytokine IL-22 [89]. While most of the studies focused on the impact of microbial interventions on the gut microbial community, Samara and colleagues [89] explored the host–microbial interactions that maintain gut homeostasis via balancing inflammatory and regulatory immune responses. Although the authors did not capture the impact of the probiotic interventions on health due to the small sample size, this study represents a crucial step in identifying microbial interventions with positive effects on host health. 

Besides the lack of understanding of the impact of microbial interventions on the host immune system/health, there are other challenges in designing successful interventions using probiotics. Most commercial probiotic organisms do not contain the required traits to utilize the resources and colonize the gut due to the absence of host specificity [90]. In order to become residents in the gut, probiotics must be supplemented in live form in sufficient quantities, overcome habitat barriers (pH, bile salts, etc.), and compete with residents for nutrients and niches via competition, antagonism, and mutualism [90]. Probiotic organisms contain these traits if they share an evolutionary relationship with host GIT [91]. Current probiotic strains used in human trials were either isolated from fermented foods or mice [92]. Moreover, the presence of host-specific microbes is vital for the proper priming of the immune system [93]. The use of humanized mice (human-microbiota-colonized mice) revealed that host-specific Firmicutes are required for the differentiation and proliferation of T-cells [94]. Moreover, humanized mice had less protection against enteric infections than conventional mice colonized with mouse microbiota [93], suggesting that probiotics for microbial interventions might also need to be acquired from the relevant host (Table 1).

In addition to probiotics, prebiotics (microbial growth substrates) facilitate the growth of beneficial microbiota. Prebiotics have been used to restore perturbed microbial communities of the neonates successfully. A study on malnourished Bangladesh infants revealed that a prebiotic mixture targeting beneficial organisms in the gut restored microbial composition and growth retardation [25]. Malnutrition is one of the factors that affect the trajectories of the colonization of microbiota in children [95]. Following the identification of microbial groups linked to malnutrition, the researchers developed substrates that promote the growth of malnutrition-related bacteria [96] and supplemented the infants during early life to restore the perturbed microbial communities [97,98]. When the prebiotic supplement restored the gut microbial composition to that of well-nourished children, the malnourished children started to recover their growth. This is one of the most significant studies revealing that microbial interventions during early life can be used to influence host physiology. Similarly, livestock models have also used prebiotics to improve gut health by modulating microbial composition. Resistant potato starch is a prebiotic that is resistant to host enzymatic digestion and can be fermented by the lower GIT microbiota [99]. Supplementing weaning pigs with resistant potato starch has been shown to increase beneficial bacteria (e.g., Bifidobacteria) and decrease diarrhea incidences [100]. Prebiotics can either indirectly affect the immune system via the modulation of gut microbiota and microbial metabolites or directly affect the immune system by activating innate immune cells and pattern recognition receptors (Table 2) [101]. Trachsel and colleagues [100] studied the association between the abundance of regulatory T cells (Treg) and short-chain fatty acids in the cecum and reported a positive association between butyrate concentration and the number of Tregs. However, there is a limited understanding of the causative effect of prebiotics on the immune system, especially regarding whether prebiotics supplementation during early life can induce immune programming. Similar to probiotics trials, it is important to test prebiotics during the window of opportunity to assess their persistent beneficial impact on the gut microbiome and host health.

The use of probiotics and prebiotics together (synbiotics) can be used to achieve health benefits through complementary or synergistic effects [6]. In the past, synbiotics have been used mainly in adults to mitigate microbiome-linked pathologies such as irritable bowel syndrome and obesity [110]. Recently, the oral supplementation of synbiotics (Lactobacillus plantarum and fructooligosaccharide) has been used to treat neonates with sepsis [111] and children with irritable bowel syndrome [112]. These studies reported a reduction in disease prevalence with synbiotics treatments. However, the supplementation of synbiotics on resident gut microbiota, homeostasis, and immune function in neonates has not been well studied. FMT and vaginal seeding are other microbial interventions used in cesarean-delivered infants; however, these interventions should be assessed in the long run to understand their impact throughout the life span. Perturbed microbial communities, due to the lack of exposure to maternal microbiota, increase the risk of developing microbiome-linked pathologies [13,14,15]. Therefore, it is necessary to evaluate the impact of these microbial interventions on the priming of the immune system during early life and on generating immune memory. Such understanding can lead to the design of successful early-life interventions with positive impacts on both gut microbiome and health. 

## 5. Future Directions and Summary

**Figure 1:**
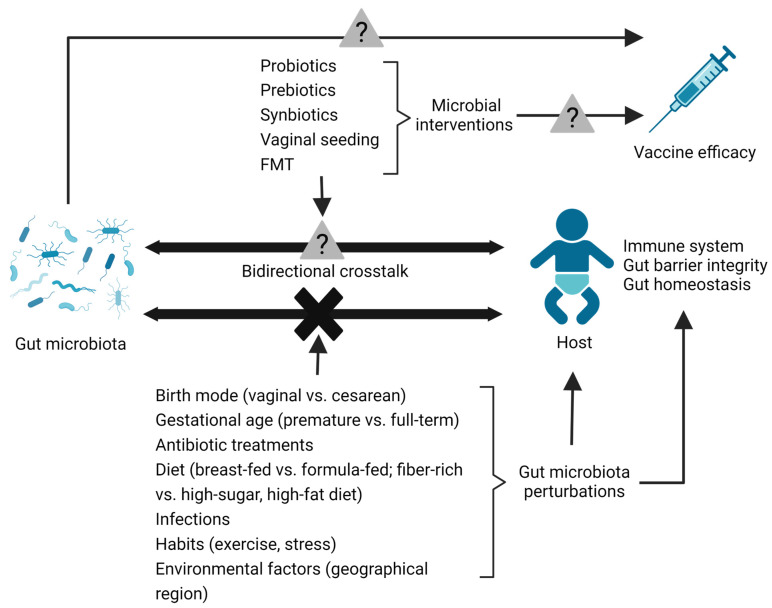
The use of microbial interventional tools to restore bidirectional communication and neonatal vaccine efficacy. Bidirectional crosstalk between host and microbiota is crucial for developing the immune system, gut barrier functions, and maintaining gut homeostasis and gut health during early life. This interaction can be affected by various internal and external factors, such as birth mode, gestational age, antibiotic treatments, diet, infections, habits, and environmental factors. In addition, early-life immunization programs minimize neonatal infections. The ability of vaccines to generate antibody responses/vaccine efficacy is highly inconsistent among different populations. One of the factors influencing vaccine efficacy is gut microbiota. Dietary/nutritional changes affect the alteration of gut microbiota and neonatal immunity. Thus, diet/nutrition indirectly influences vaccine efficacy by altering the gut microbiota. Microbial interventions in early life can improve gut health by restoring perturbed gut microbiota, host–microbial communication, and neonatal vaccine efficacy (?—future research are required to assess the direct impact of gut microbes and early life microbial interventions on host responses to vaccination).

Recent research has enhanced our understanding of the bidirectional communication between microbiota and the host (Figure 1). This is a vital process for the developing immune system, gut barrier function, and gut homeostasis during the neonatal period. Thus, the neonatal period provides a window of opportunity to intervene in gut microbiota and to create a beneficial impact on the host. With the increasing prevalence of microbiome-linked pathologies in humans and the need for alternatives to antimicrobials in livestock production systems, there is a tremendous opportunity to design successful microbial interventions to improve gut health during early life (Figure 1). Well-designed, randomized, controlled trials can be used to evaluate the efficacy of early-life microbial interventions to reduce the prevalence of infections or microbiome-linked pathologies. In addition, such trials in animal models are vital to understanding the mechanisms with which these microbial interventions prime immune memory, with long-term effects.

Besides understanding the window of opportunity, knowledge of host-specific microbial priming of the immune system is an important turning point for research aiming to develop microbial interventions. Recent microbiome research in human and other animal models has successfully generated an in-depth understanding of microbial communities and their functions. These studies provide the basis for identifying host-specific microbial groups and microbial groups linked to host physiology and immunology. This is one of the reasons behind the development of successful prebiotic supplements as a microbial intervention to mitigate malnutrition-related growth retardation [82,83]. Similar collective efforts among researchers working on microbiome, nutrition, physiology, and immunology will be essential in the future for designing successful interventions to improve gut health and overall health in the long term. 

## Figures and Tables

**Table 1:** Limitations of the current microbial interventions and suggestions for future research.

Limitations	Potential Suggestions for Future Research
Mainly focus on changes in health status [87]	Assessment of microbial community profiles, immune modulation, and gut barrier integrity
Use stool samples to identify gut microbiota [88]	Assessment of regional gut microbial communities
Use digesta samples to identify microbial communities [61]	Assessment of both tissue- and digesta-associated microbial communities
Non-host probiotics (e.g., fermented food and mice) [92]	Host-derived/specific probiotic strains
Use of allochthonous organisms (passengers) as probiotics [93]	Use of autochthonous organisms (colonizers) as probiotics

**Table 2:** Mechanisms of probiotics and prebiotics in regulating gut microbiota, the immune system, and gut barrier integrity.

	Probiotic	Refs.	Prebiotic	Refs.
Shaping Gut Microbial Community	Prevent the colonization of pathogens via colonization resistance.	[102]	Are utilized by the specific beneficial organisms that reside in the gut and enhance the population of beneficial microbes.	[103]
	Are resistant to intestinal pH and bile salt and colonize the gut by competing with pathogens (e.g., producing bile salt hydrolases and EPS by Bifidobacterium).	[104]	Provide an energy source for the gut microbiota and produce metabolites (e.g., SCFAs) to assist the growth and function of other microbiota (cross-feeding).	[101]
	Compete for the nutrients and niches with pathogens and prevent the colonization of opportunistic pathogens (spatial and nutritional competition).	[102]	Present antagonism against pathogens by increasing the beneficial microbial growth.	[105]
	Produce metabolites (short-chain fatty acids—SCFAs) that can be utilized by the resident microbes (e.g., cross-feeding).	[101]	Contain anti-adhesive properties against opportunistic pathogens.	[106]
	Capable of utilizing the available resources through enzymatic metabolism (e.g., carbohydrate utilization enzymes in Bifidobacterium allow it to utilize oligosaccharides present in human milk).	[104]		
Maintaining Gut Barrier Integrity and Immune Modulation	Allow for immunostimulation or immunoregulation.	[102]	The fermentation of prebiotics produces SCFAs, which modulate the immune signaling via the expression of cytokines and innate immune cells.	[107]
	Secrete antimicrobial peptides to prevent the attachment of pathogens to epithelial cells.	[102]	Favor the colonization of beneficial microbes that promote homeostasis.	[102]
	Promote mucin production.	[108]	Directly influence the expression of pattern recognition receptors on epithelial cells.	[101]
	Increase the production of tight-junction proteins and reduce bacterial translocation.	[102]	Interact with carbohydrate receptors on immune cells.	[102]
	Regulate immune cell activation.	[102]	Modulate the expression of anti- and pro-inflammatory cytokines.	[102]
	Act as a barrier against scavenging agents like reactive oxygen species during inflammation.	[109]		
	Crosstalk with the host immune system through metabolites like SCFAs and cell wall components like EPS.	[109]		

## Data Availability

No new data were created or analyzed in this study. Data sharing is not applicable to this article.
